# The adaptation of Turkish health literacy scale for literate Syrian adult refugees living in Turkey: a reliability–validity study

**DOI:** 10.1186/s13031-021-00401-5

**Published:** 2021-08-28

**Authors:** Şevkat Bahar Özvarış, Bahar Güçiz Doğan, Hande Konşuk Ünlü, Gamze Aktuna, Tacettin İnandı, A. Öner Kurt, Neriman Aydın, M. Tezer Kutluk

**Affiliations:** 1 Public Health Institute, Hacettepe University, Ankara, Turkey; 2 Research for Health in Conflict in the Middle East and North Africa (R4HC-MENA), and the Conflict and Health Research Group (CHRG), King’s College, London, UK; 3 Department of Public Health, Faculty of Medicine, Hacettepe University, Ankara, Turkey; 4 Department of Public Health, Faculty of Medicine, Mustafa Kemal University, Hatay, Turkey; 5 Department of Public Health, Faculty of Medicine, Mersin University, Mersin, Turkey; 6 Department of Public Health, Faculty of Medicine, Gaziantep University, Gaziantep, Turkey; 7 Department of Pediatric Oncology, Faculty of Medicine and Cancer Institute, Hacettepe University, Ankara, Turkey

**Keywords:** Health literacy scale, Scale adaptation, Reliability–validity, Syrian refugees, Confirmatory factor analysis, Cronbach’s alpha

## Abstract

**Background:**

Turkey hosts the world’s largest refugee population of whom 3.5 million are Syrians and this population has been continuously growing since the year 2011. This situation causes various problems, mainly while receiving health-care services. In planning the migrant health-care services, for the policy makers of host countries, health literacy level of migrants is an important measure. Determination of health literacy level of Syrian refugees in Turkey would be supportive for planning some interventions to increase health-care service utilization, as well as health education and health communication programs. An “original health literacy scale” for 18–60 years of age Turkish literate adults (Hacettepe University Health Literacy Scale-HLS) was developed to be used as a reference scale in 2018. Since it would be useful to compare the health literacy levels of Turkish adults with Syrian adult refugees living in Turkey with an originally developed scale, in this study, it was aimed to adapt the HLS-Short Form for Syrian refugees.

**Methods:**

This methodological study was carried out between the years 2019–2020 in three provinces of Turkey where the majority of Syrians reside. The data was collected by pre-trained, Arabic speaking 12 interviewers and three supervisors via a questionnaire on household basis. At first, the original Scale and questionnaire were translated into Arabic and back translated into the original language. The questionnaire and the Scale were pre-tested among 30 Syrian refugees in Ankara province. A total of 1254 refugees were participated into the main part of the study; 47 health-worker participants were excluded from the validity-reliability analysis. Confirmatory factor analysis (CFA) was performed. Cronbach’s alpha and Spearman–Brown coefficients were calculated.

**Results:**

Of the participants, 52.9% was male; 26.1% had secondary education level or less; almost half of them had moderate economic level; 27.5% could not speak Turkish. The Cronbach’s Alpha was 0.75, Spearman–Brown Coefficient was 0.76; RMSEA = 0.073, CFI = 0.93, TLI = 0.92 and GFI = 0.95 for the Scale. The Cronbach’s Alpha was 0.76, Spearman–Brown Coefficient was 0.77; RMSEA = 0.085, CFI = 0.93, TLI = 0.91 and GFI = 0.95 for self-efficacy part.

**Conclusion:**

In conclusion, the adapted HLS would be a reliable instrument to evaluate the health-literacy level of Syrian refugees living in Turkey and could allow for a comparison of the host country’s health literacy level to that of the refugees using the same scale.

## Background

Health literacy is defined as “the degree to which individuals have the capacity to obtain, process, and understand basic health information and services needed to make appropriate health decisions” [1]. In addition, health literacy represents “the cognitive and social skills which determine the motivation and ability of individuals to gain access to, understand and use information in ways which promote and maintain good health” [2]. Many researches have mentioned the impact of health-literacy on health outcomes as well as on preventive, curative and rehabilitative health care [3, 4]. According to a WHO document on health promotion, “health literacy promotes empowerment, which in turn is vital for achieving the internationally agreed health and development goals as well as the emerging threats such as from the pandemic influenza, climate change and non-communicable diseases” [5].

Insufficient health literacy is highly related to reduced access to health services, trouble in managing health problems, limited understanding of health-related information and ability in making logical decisions interrelated health issues [6]. In societies, improving health literacy provides a substructure that enables individuals to play an active role in improving their health, to successfully engage in their social duties for health, while compelling governments to fulfill their responsibilities to address health and health equity [7, 8].

Migration involves a much deeper adaptation mechanism than individual adjustments to new environments and is in a negotiation process with social, political and economic forces. In this context, immigration should be considered as a fundamental social determinant of health, since it is a lived experience that directly affects health and well-being [9]. The language barrier for immigrants poses a major problem in health care services related situations like seeking and receiving appropriate health services [10, 11]. The results of a study conducted in Switzerland, which compared indigenous people with immigrants show that the immigrants do not carry their health literacy knowledge to the new homeland, even if it affects their health status in long term. However, in the medical situations, the language barrier can become more important in the short term and can prevent immigrants from seeking and receiving appropriate health services [11]. Health literacy is significantly and consistently associated with the quality of care, and immigrants with adequate health literacy in reading hospital documents reported higher level of quality of medical care compared to immigrants with poor health literacy [12].

Immigrants are transported to host countries along with a range of different health beliefs, cultural behaviors, and previous experiences, which ultimately affect immigrants’ health outcomes at every point of interaction with the new health-care system [13]. Cultural beliefs about health and illness are integral to a patients’ ability to understand and act on their doctor’s instructions [14].

It would not be wrong to argue that immigrants are affected more by all other social determinants of health than the citizens of the country they migrate to. In a study conducted in Canada on immigrants, education level, employment status and income were the mediators for health literacy and disability relationship, and it was stated that improving health literacy benefits everyone, but that individuals with less than a high school education and who are more likely to be unemployed will gain the most [15]. This context reveals how important health literacy studies are for immigrants.

After opening the borders of Turkey to Syrians, since 2011, the number have increased 3.6 million registered refugees. Great amount of refugees lives mostly in city centers, with the highest numbers in Istanbul, Gaziantep, Şanlıurfa and Hatay provinces [16]. Migrants with legal status could utilize Migrant Health Centers (MHC) and Migrant Health Units (MHU) in order to obtain primary health care services (PHC) free of charge provided by the Turkish Government (175 MHCs and 785 MHUs were established in 29 cities in Turkey). As of August 2020, 2520 Syrian health personnel and 966 patient referral guides (translators) had been trained and have been providing services in MHCs/MHUs across the country [17].

The continuing growth of refugee population in Turkey, has resulted in some adaptation problems. According to some studies, problems stated by the migrants while obtaining health services were lack of trust, fear of health-care personnel, lack of health insurance, lack of communication and consequently not being able to give informed consent, and inability to control privacy [18–20]. Due to language barrier, refugees may be less likely to benefit from health services which might affect the adherence to the treatment [21]. In some recent studies, it was suggested that migrants utilize health services less compared with the host community [22].

At the end, limited health literacy of immigrants provokes ineffectual use of health-care resources, resulting with increased personal and public expenditures. Health literacy level of migrants is an important indicator for the policy makers of host countries in planning the migrant health services. Determination of health literacy level of Syrian refugees in Turkey would be supportive for health service utilization as well as health education and health promotion programs that will change the health behaviors.

Health literacy scales are important tools for assessing the health literacy level. There are significant differences in which dimensions are taken into consideration regarding health literacy [23, 24]. In different papers, health literacy defined as a combination of skills including the ‘print literacy’, ‘numeracy or quantitative literacy’ and ‘oral literacy’ [25, 26]. In other respect, most widely discussed approaches to literacy classification included “functional, interactive, and critical literacy” levels. This comprehensive approach indicated that the different levels of literacy progressively allowed for greater autonomy and personal empowerment [27, 28]. TOFHLA evaluates the reading comprehension and numeracy [29]; REALM identifies the patient’s reading skills [30]; REALM-R is only a word recognition test [31]; Newest Vital Sign (NVS) test, assesses reading and interpretation skills [32]. There are some studies, which performed the Arabic language adaptation processes related to some of the health literacy scales [33–35].

An “original health literacy scale” for Turkish literate adults between 18 and 60 years of age (Hacettepe University Health Literacy Scale-HLS) was developed to be used as a reference scale in 2018 [36]. After developing the 71-item long form, a short form (24 health literacy related items + 16 self-efficacy statements) was also validated. The analysis showed that the Scale could be used as a reference scale to assess the health literacy level for Turkish literate adults. This scale includes items related to the three levels of cognitive domain of Bloom’s Taxonomy (“knowledge”, “comprehension” and “application”) as well as the self-efficacy statements, based on the affective domain. At the same time, original Turkish HLS assesses “disease prevention and health promotion”, and “treatment and access to health services” within the aforementioned context [36].

It is important to use a standard tool when it is aimed to compare health literacy levels of different communities. From this perspective, using an original health literacy scale developed for the host community would be more appropriate to compare the level of health literacy of Syrian refugees’ and native Turkish people. The cultural similarities between the Syrian and Turkish people derived from history, and the existence of several similar Arabic and Turkish words in the two languages motivated the authors to adapt the originally developed Turkish HLS short form into Syrian Arabic.

## Methods

### Participants and procedure

This methodological study was carried out in Hatay, Mersin and Gaziantep provinces where the Syrians mostly live in Turkey between the years 2019 and 2020. Data was collected by 12 previously trained Arabic-speaking interviewers (half female) with the supervision of three academicians. The validity-reliability study was carried out on the Syrian refugees in the same age group. The data were collected via a questionnaire (including questions related to some socio-demographical characteristics and HLS) on household basis. Approximately 400 Syrian refugees, equal number from each gender in each age group (18–29, n = 459, 38%; 30–44, n = 422, 35%; 45–60, n = 326, 27%) were randomly recruited from each province (Gaziantep n = 382, Hatay n = 431, Mersin n = 395). A total of 1254 refugees were participated in the study. Forty-seven health worker participants were excluded from the validity-reliability analysis in order not to ruin the results as was done in the validity analysis of the original scale. Final analysis was performed with 1207 participants.

Other health literacy scales often used in the literature do not include the same Bloom’s taxonomy dimensions on which the validated original Turkish HLS was developed. Criterion validity was assessed according to an established criterion determined by the researchers (that is comparing with the scores of health personnel) [37]. In this study, the criterion is that health worker participants had a higher health literacy level. For this reason, the scores of 47 health workers not included in the validity-reliability analysis were used to assess the criterion validity of the adopted HLS. Criterion validity is the difference between the mean of measures of a group expected to perform poorly or high and a group that should perform normally. When there is a difference in favor of the group expected to show low or high performance, the criterion validity of the scale is ensured [38, 39]. Bannighan and Watson stated that “It is important to be sure that the gold standard is a true gold standard in terms of its psychometric properties and not just a scale that is in common usage but has no reliability or validity” [40]. Based on this information, from the study group of 1207 non-health worker participants, 47 were randomly selected from the same age and sex groups together with the health worker participants in the present study. The mean scores of the scale and self-efficacy part of health-worker and non-health worker participants were compared for the purpose of criterion validity analysis.

### Instruments

The short form of the HLS-Turkish version, which adapted via this study consisted of two parts: A 24-item knowledge-based health literacy part (one dimension) and a 16-statement self-efficacy part. In the health literacy section, each item was given a score of “1” for correct answers and “0” for incorrect answers. The scores range from 0 to 24, with 0 being the lowest and 24 being the highest. The scale’s self-efficacy section is also one-dimensional. The statements were scored as 1: never, 2: sometimes, 3: always. The minimum-maximum scores could be obtained are 16-48. Due to the different scoring system of the parts, a total score could not be calculated.

The reliability–validity results showed that HLS-Short Form is a valid and reliable tool. The reliability results of the Health Literacy Scale are as follows: Cronbach’s alpha = 0.84 for internal consistency and Spearman–Brown = 0.78 for split-half reliability; Root Mean Square Error of Approximation (RMSEA) = 0.049, Goodness of fit (GFI) = 0.94, Adjusted Goodness of Fit (AGFI) = 0.93 and Normed Fit Index (NFI) = 0.94. The reliability results of the Self-Efficacy part are Cronbach’s alpha = 0.83 and for internal consistency and Spearman–Brown = 0.73 for split-half reliability; RMSEA = 0.068, GFI = 0.94, AGFI = 0.91 and NFI = 0.94 [36].

### Statistical analysis

Data entry and evaluation were conducted through statistical package program IBM SPSS 23.0. The validity and reliability analysis were performed using The R Project for Statistical Computing (ver. 4.0.0) program. Confirmatory factor analysis (CFA) based on polychoric correlations diagonally was applied to confirm the factor structure of the scale. Diagonally weighted least squares (DWLS) method was used to get more accurate parameter estimates. In addition, modification indices were also obtained. If needed, the correlation between error terms was added to the model according to high modification indices. In order to demonstrate the reliability, the Cronbach’s alpha reliability coefficient, which shows internal consistency, and Spearman–Brown coefficients, which shows the two-half reliability, were calculated. The difficulty and discrimination coefficients and Cronbach’s alpha if item deleted statistics calculated when the item was deleted. To demonstrate criterion validity, the average scores of Health-Literacy part and Self-Efficacy part of the health workers and non-health workers were compared via independent samples t test; $p<0.05$ was accepted as significance level.

## Results

A total of 1315 refugees were invited to participate in the study, 1254 refugees were participated voluntarily with a participation rate of 95.4% (47 of them were health workers and 1207 were non-health workers; the analysis was performed on non-health workers mainly). Among the participants (n = 1207), 52.9% were men; almost half of them were college/university graduates; 60.7% were married and living with their spouse; 47.6% were currently working; a quarter indicated that their economic situation was below average or poor. Ninety-eight point four percent of the participants said their native language was Arabic, 1.5% said Kurdish or Turkish, and 27.5% said they didn’t speak Turkish. One-fifth of the participants had at least one chronic disease and 48.2% had health insurance (Table 1).

**Table 1:** Some characteristics of participants (Turkey, 2019)

Characteristics	n^a^	%
*Sex (n = 1207)*		
Male	639	52.9
Female	569	47.1
*Educational status (n = 1200)*		
Primary school	80	6.7
Secondary school	233	19.4
High school	297	24.8
University	590	49.2
*Marital status (n = 1205)*		
Never married	386	32.0
Married and living with spouse	731	60.7
Widowed/divorced/separated	88	7.4
*Working status in Turkey (n = 1206)*		
No	628	52.4
Yes	573	47.6
*Economic status (n = 1205)*		
Very good	57	4.7
Good	278	23.1
Moderate	566	47.0
Below moderate	203	16.8
Poor	101	8.4
*Speaking Turkish (n = 1202)*		
No	330	27.5
Moderate	493	41.0
Yes	379	31.5
*Health insurance (n = 1186)*		
No	604	50.1
Yes	582	48.3
*Having any chronic disease (n = 1203)*		
No	951	79.1
Yes	252	20.9

^a^There are various number of missings for every variable

### Adaptation process of the scale

#### Language validity

As the first stage, the Scale and questionnaire were translated into Arabic by an expert whose native language is Syrian Arabic and who is fluent in Turkish. Another expert whose mother tongue is Arabic and speaks Turkish fluently made the back translation. The back translated Scale was compared with the original Scale by the research team and Turkish Language experts, and the process of Arabic translation of the Scale was completed.

An Arabic speaking interviewer pretested the questionnaire and the first draft of the Scale on 30 Syrian refugees from different age groups (18–29, 30–44, 45–60) in Ankara Province. The results of the pilot study were evaluated by the research team in collaboration with the Arabic–Turkish translators and the final version of the Scale was obtained.

### The validity–reliability results of health literacy part of the scale

#### Item analysis results

Cronbach’s alpha statistics when item deleted, difficulty and discrimination values of items were obtained. The difficulty levels of items ranged between 0.29 and 0.93. All of the items were positively correlated with the total score and ranged between 0.14 and 0.69. Item 9 and Item 12 were excluded by taking experts’ opinions, since item-total correlation were less than 0.20. After that, reliability analysis was performed again and the item statistics for remaining items were given in Table 2. According to item analysis results, difficulty levels of items varied between 0.29 and 0.93 and item-total correlation values varied between 0.22 and 0.72 for the 22-item one-dimensional Health Literacy part of the Scale.

**Table 2:** Item statistics and reliability values of health literacy part of the scale

	Difficulty	Discrimination values (point biserial correlation)	Cronbach’s alpha (when item deleted)
Item 1	0.93	0.37	0.75
Item 2	0.84	0.46	0.74
Item 3	0.93	0.31	0.75
Item 4	0.76	0.45	0.74
Item 5	0.67	0.48	0.74
Item 6	0.72	0.42	0.74
Item 7	0.87	0.56	0.74
Item 8	0.89	0.56	0.74
Item 10	0.76	0.52	0.74
Item 11	0.55	0.36	0.75
Item 13	0.4	0.29	0.75
Item 14	0.77	0.22	0.75
Item 15	0.93	0.72	0.74
Item 16	0.79	0.51	0.74
Item 17	0.29	0.25	0.75
Item 18	0.85	0.6	0.74
Item 19	0.91	0.45	0.75
Item 20	0.8	0.7	0.73
Item 21	0.78	0.68	0.73
Item 22	0.73	0.61	0.73
Item 23	0.78	0.26	0.75
Item 24	0.74	0.22	0.76
Cronbach’s alpha		0.75	
Spearman–Brown coefficient		0.76	

The internal consistency was 0.75, which showed a high level of reliability. The Spearman–Brown coefficient result showed that split-half reliability was sufficient.

#### Confirmatory factor analysis results

**Fig. 1:**
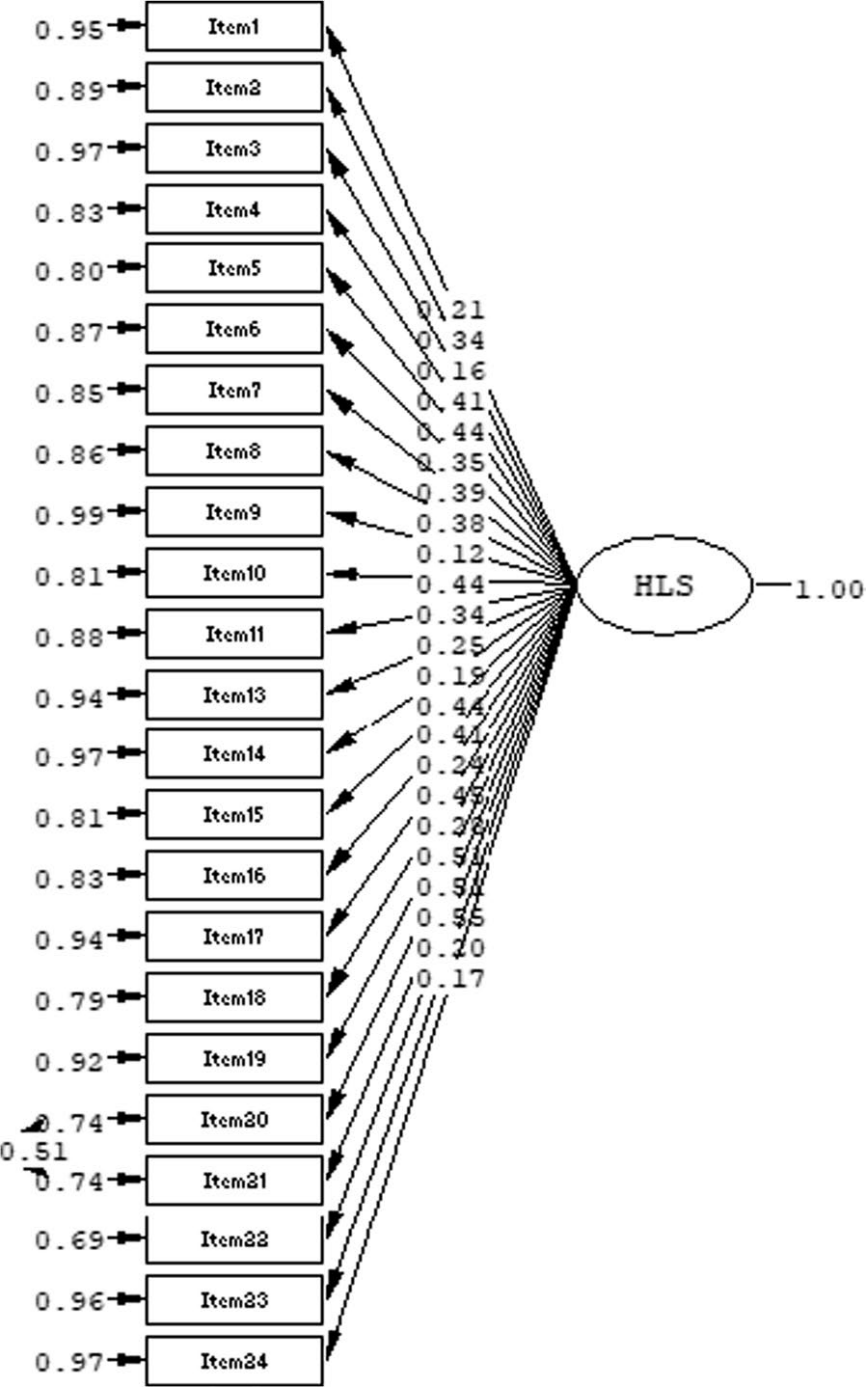
The path diagram of health literacy part of the scale

In order to confirm the construct validity, confirmatory factor analysis (CFA) based on polychoric correlations was applied since there was a theoretical basis for one-dimensionality with 22 items, which was formed by item analysis and experts’ opinions. The path diagram was given in Fig. 1.

As seen in Fig. 1, standardized factor loadings ranged between 0.12 and 0.55. As the fit indices, RMSEA was 0.083, Comparative Fit Index (CFI) was 0.91, Tucker–Lewis Index (TLI) was 0.90 and GFI was 0.93. According to the modification indices, the results suggested to add correlation between the error terms of Item 20 and Item 21. After taking experts’ opinions, it was decided that this modification was appropriate. The analysis was repeated again and fit indices were calculated. The RMSEA was found as 0.073 which reflects a good fitness. The other fit indices were CFI = 0.93, TLI  =  0.92 and GFI = 0.95. The results demonstrated that model had a very good fitness and the construct validity was confirmed.

### The validity–reliability results of self-efficacy part of the scale

#### Item analysis results

Cronbach’s alpha statistics when item deleted, difficulty and discrimination values of items were obtained. All of the items were positively correlated with the total score and ranged between 0.12 and 0.62. Item 14 was excluded by taking experts’ opinions, since item-total correlation was less than 0.20. Reliability analysis was performed again and the item statistics for remaining items were given in Table 3. As seen from the table, item-total correlation values of Item 15 and Item 16 were less than 0.20. After taking experts’ opinions, it was decided to keep these items that they could negatively affect the content validity if excluded. Item-total correlation values varied between 0.12 and 0.63 for the one-dimension Self-Efficacy part of the Scale with 15 items.

**Table 3:** Item statistics and reliability values of self-efficacy part of the scale

	Difficulty	Discrimination values (point biserial correlation)	Cronbach’s alpha (when item deleted)
Item 1	2.02	0.48	0.74
Item 2	2.02	0.60	0.73
Item 3	1.75	0.54	0.74
Item 4	2.18	0.60	0.73
Item 5	1.80	0.63	0.73
Item 6	2.30	0.31	0.76
Item 7	2.30	0.61	0.73
Item 8	2.49	0.33	0.76
Item 9	2.04	0.53	0.74
Item 10	1.86	0.27	0.76
Item 11	1.92	0.49	0.74
Item 12	2.33	0.21	0.76
Item 13	2.12	0.26	0.76
Item 15	2.58	0.17	0.77
Item 16	2.53	0.12	0.77
Cronbach’s alpha		0.76	
Spearman–Brown coefficient		0.77	

The internal consistency was 0.76, which signed a high level of reliability. The Spearman–Brown coefficient showed that split-half reliability was sufficient.

#### Confirmatory factor analysis results

**Fig. 2:**
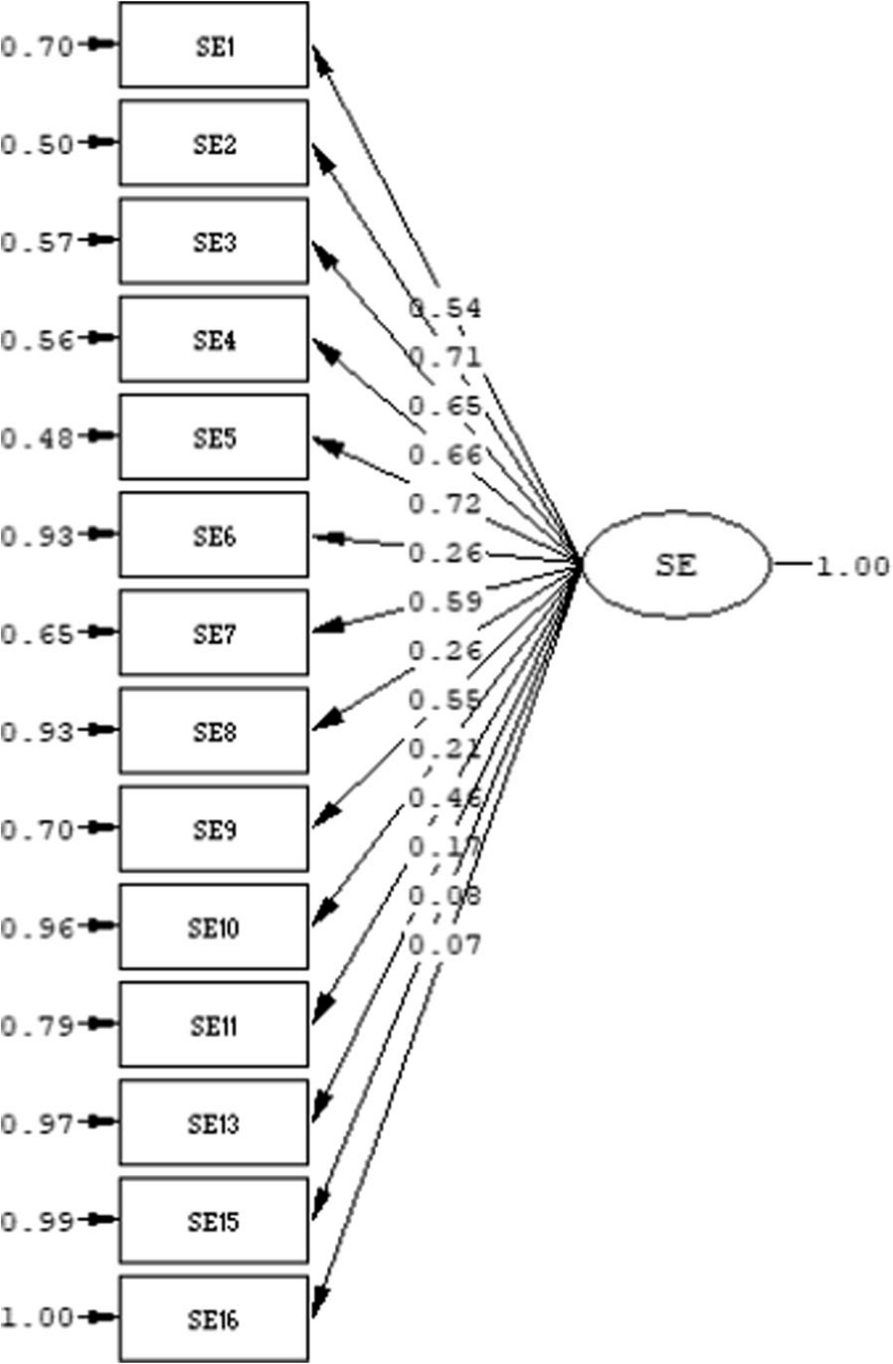
The path diagram of self-efficacy part of the scale

In order to confirm the construct validity, confirmatory factor analysis (CFA) based on polychoric correlations was applied since there was a theoretical basis for one-dimensionality with 15 items, which was formed by item analysis and experts’ opinions. The path diagram was given in Fig. 2.

Standardized factor loadings ranged between 0.07 and 0.72 (Fig. 2). The fit indices were as follows: RMSEA = 0.085, CFI = 0.93, TLI = 0.91 and GFI = 0.95. The model had a very good fitness and the construct validity was confirmed.

### Criterion validity

In order to demonstrate criterion validity, the mean scores of the scale and self-efficacy part of health-worker and non-health worker participants were compared. The summary statistics were given in Table 4.

**Table 4:** The summary statistics of the scores for health and non-health workers

	Health literacy part scores		Self-efficacy part scores	
	Non-health workers	Health workers	Non-health workers	Health workers
$\text {Mean} \pm \text {SD}$	$16.36\pm 3.90$	$18.72\pm 2.80$	$32.66\pm 5.69$	$35.2\pm 5.81$
$\text {Median}$	18	20	33	37
$Q_{1}$–$Q_{3}$	14.0–19.0	17.0–21.0	30.0–37.0	31.0–39.0
Min–Max	3–21	13–22	20–42	22–44
$\text {Count}$	47	47	47	47
***p-value***	**0.005**^a^		**0.041**^a^	

*SD* standard deviation, $Q_{1}$: 1st quartile, $Q_{3}$: 3rd quartile, *Min* minimum, *Max* maximum

^a^Independent Samples t-test result

The mean total Health Literacy part scores for non-health workers and health workers were $16.36\pm 3.90$ and $18.72\pm 2.80$, respectively. The mean total Self-Efficacy part scores for non-health workers and health workers were $32.66\pm 5.69$ and $35.2\pm 5.81$, respectively. When the groups were compared in terms of Health Literacy and Self-Efficacy scores, statistically significant difference was found between health and non-health workers. Moreover, the Pearson correlation coefficients were calculated for the scale and self-efficacy part separately between two groups ($r=0.136$, $p=0.363$ for the scale; $r =-0.005$, $p=0.976$ for self-efficacy part), that indicates the adapted scale had a good criterion validity.

## Discussion

The Cronbach’s alpha value of the health literacy part of the scale was found as 0.75, and self-efficacy part was 0.76 for Syrian refugees which was a little lower than Turkish version of HLS (0.84 and 0.83, respectively) [36].

Al-Jumaili et al. [33] performed an Arabic adaptation of short version of TOFHLA and NVS, in which they found Cronbach’s alpha value for reading section of S-TOFHLA as 0.89, numeric questions as 0.62. The Cronbach’s alpha of NVS was 0.69 [33].

Another study performed in Sweden on adult asylum seekers, Swedish Functional Health Literacy Scale (S-FHL) and the short European Health Literacy Questionnaire (HLSEU-Q16) were translated into Arabic. Cronbach’s alphas were calculated to explore the internal consistency of the questions used for quality of communication and receiving health care information. The internal consistency values were found as = 0.79 and 0.71, respectively) [34].

The health literacy level of Syrian refugees living in Turkey was evaluated in a report published by WHO [41], in which same scales that Wångdahl et al. [34] used. One of the scales used, HLSEU-Q16, measures comprehensive health literacy, while the S-FHL measures functional health literacy. The researchers considered using the original Arabic versions of the S-FHL and the HLS-EU-Q16 in this study, nevertheless according to the researchers “the Arabic was not a good match for the Syrian dialect of common Arabic. Therefore, Syrian Arabic versions were developed, pretested and validated before implementation”, however there is no information given about the validation process and measures in the report.

Another study conducted to quantify current health literacy levels amongst a segment of the Syrian refugee population in Canada by translating and validating an existing comprehensive health literacy assessment tool, the All Aspects of Health Literacy Scale (AAHLS) into Arabic with a Cronbach’s alpha of 0.67 for the overall scale and 0.63 for communicative items [35].

Thus, the reliability–validity results of the present study showed that the adopted scale is a valid and reliable tool to assess health literacy levels of Syrian refugees resided in Turkey, with similar values of other Arabic language adapted health literacy scales.

Before planning various health services, training and promotion activities for Syrian refugees in Turkey, evaluation of the HLS level of these people might be very useful by using this validated scale. This information would be a valuable input for Ministry of Health and, international and national agencies (which provide some sort of health services) for planning processes as well as monitoring the refugee health services.

Given that, almost half of the participants of this study were university graduates, it would be better to renew the validation process of the scale on literate Syrian refugees, with lower educational levels. Likewise, 40% of the female Syrian refugees and 35% of males were illiterate according to the results of 2018 Turkey Demographic and Health Survey—Syrian Sample [42]. Due to this fact, it is suggested that a new version of adapted scale be developed.

### Limitations

The current study has some limitations: Even the participants were recruited from three provinces of Turkey, due to the sampling method (convenience sampling), the external validity of the results was found to be weak. Due to the high mobility of the refugees, as it is extremely difficult to identify the same people at the same address even after a week it is impossible to re-visit a sub-sample of the group for test-re-test purpose. Almost half of the participants of this study were university graduates. The participants of the study were literate 18–60 aged adults. For this reason, it is not appropriate to use this scale for assessing the health literacy level of adolescents and elderly people. Since the scale can only be self-administered, it is not suitable to use it for the assessment of illiterate people.

## Conclusion

In conclusion, the reliability–validity analysis showed that the adopted scale is a valid and reliable tool to assess health literacy levels of Syrian refugees resided in Turkey. Assessing the health literacy level of Syrian refugees living in Turkey via this reliable scale will contribute to health policy formulation as well as planning various activities such as specific health-care services and trainings. Moreover, it would be valuable to assess health literacy level of refugees in the other countries with same scales used for evaluating the health literacy level of native people mentioned in some studies.

## Data Availability

The data sets generated and/or analyzed during the current study are not publicly available for now since this study comprises one part of a comprehensive project and other stages are not yet completed, but are available from the corresponding author on reasonable request.
